# Administration of misoprostol by trained traditional birth attendants to prevent postpartum haemorrhage in homebirths in Pakistan: a randomised placebo-controlled trial

**DOI:** 10.1111/j.1471-0528.2010.02807.x

**Published:** 2010-12-23

**Authors:** N Mobeen, J Durocher, NF Zuberi, N Jahan, J Blum, S Wasim, G Walraven, J Hatcher

**Affiliations:** a Department of Community Health Sciences, Aga Khan UniversityKarachi, Pakistan; b Gynuity Health ProjectsNew York, NY, USA; c Department of Obstetrics and Gynaecology, Aga Khan UniversityKarachi, Pakistan; d Aga Khan Health ServicesChitral, Pakistan; e Delegation of the Aga Khan Development Network FoundationAiglemont, Gouvieux, France

**Keywords:** Misoprostol, postpartum haemorrhage, traditional birth attendants

## Abstract

**Objective:**

To determine if misoprostol is safe and efficacious in preventing postpartum haemorrhage (PPH) when administered by trained traditional birth attendants (TBA) at home deliveries.

**Design:**

A randomised, double-blind, placebo-controlled trial.

**Setting:**

Chitral, Khyber Pakhtunkhwa Province, Pakistan.

**Population:**

A total of 1119 women giving birth at home.

**Methods:**

From June 2006 to June 2008, consenting women were randomised to receive 600 μg oral misoprostol (*n* = 534) or placebo (*n* = 585) after delivery to determine whether misoprostol reduced the incidence of PPH (≥500 ml).

**Main outcome measures:**

The primary outcomes were measured blood loss ≥500 ml after delivery and drop in haemoglobin >2 g/dl from before to after delivery.

**Results:**

Oral misoprostol was associated with a significant reduction in the rate of PPH (≥500 ml) (16.5 versus 21.9%; relative risk 0.76, 95% CI 0.59–0.97). There were no measurable differences between study groups for drop in haemoglobin >2 g/dl (relative risk 0.79, 95% CI 0.62–1.02); but significantly fewer women receiving misoprostol had a drop in haemoglobin >3 g/dl, compared with placebo (5.1 versus 9.6%; relative risk 0.53, 95% CI 0.34–0.83). Shivering and chills were significantly more common with misoprostol. There were no maternal deaths among participants.

**Conclusions:**

Postpartum administration of 600 μg oral misoprostol by trained TBAs at home deliveries reduces the rate of PPH by 24%. Given its ease of use and low cost, misoprostol could reduce the burden of PPH in community settings where universal oxytocin prophylaxis is not feasible. Continual training and skill-building for TBAs, along with monitoring and evaluation of programme effectiveness, should accompany any widespread introduction of this drug.

**Trial registration:**

http://clinicaltrials.gov/NCT00120237 Misoprostol for the Prevention of Postpartum Hemorrhage in Rural Pakistan.

## Introduction

Postpartum haemorrhage (PPH) continues to be the leading single direct cause of maternal mortality worldwide.^1^ The contribution of PPH to maternal death is disproportionately higher in developing countries, particularly in rural settings with limited infrastructure and availability of trained delivery attendants and uterotonic agents for management of PPH.^2^,^3^ Despite global efforts to ensure that women deliver with skilled birth attendants and have access to conventional uterotonics for PPH prevention, 60% of births in low-resource countries occur outside health facilities without a skilled attendant.^4^ In Pakistan, 65% of births occur at home and 27% of maternal deaths are attributed to PPH.^5^

Active management of the third stage of labour (AMTSL) is composed of immediate administration of a uterotonic, controlled cord traction for placental delivery, and uterine massage; and is internationally recognised as an evidence-based intervention that reduces PPH caused by uterine atony by up to 60%.^6^ The World Health Organization, as well as other international agencies, recommends that AMTSL be offered to all women delivering with a skilled birth attendant.^7^–^9^ Published studies comparing the efficacy and safety of various uterotonics confirm that oxytocin is the preferred drug for AMTSL.^10^–^12^ However, it is not always feasible to administer oxytocin in resource-poor settings given its requirements for cool storage, sterile equipment, skilled personnel and parenteral administration.^13^ Oxytocin prophylaxis is therefore mostly limited to facility-based deliveries and to those attended by a skilled provider, where the cold chain can be maintained, leaving the majority of deliveries in community settings with no uterotonic coverage.

Misoprostol, a prostaglandin E_1_ analogue that induces strong uterine contractions, has been explored for preventing PPH in settings where injectable uterotonics are not yet available or feasible to use. Three community-based randomised controlled trials, where misoprostol was administered at homebirths or at primary healthcare centres, have demonstrated safe and effective use of misoprostol for PPH prevention.^14^–^16^ A study in India confirmed that administration of 600 μg oral misoprostol after delivery of the baby significantly decreased the occurrence of PPH (≥500 ml) (relative risk [RR] 0.53, 95% CI 0.39–0.74).^16^ A study in the Gambia comparing oral misoprostol (600 μg) with standard care (2 mg oral ergometrine) administered by trained traditional birth attendants (TBAs) at homebirths, showed a nonsignificant trend in reduction of PPH with misoprostol, and a statistically significant smaller drop in haemoglobin (Hb) in the misoprostol arm.^14^ The third trial, testing a 600 μg regimen of sublingual misoprostol administered by midwives in primary healthcare centres in Guinea-Bissau, found that misoprostol was significantly better than placebo in reducing severe PPH ≥ 1000 ml (11% misoprostol versus 17% placebo).^15^ A meta-analysis of the three trials shows a statistically significant reduction in blood loss ≥1000 ml (2% misoprostol versus 6% control).^12^

In 2007, the World Health Organization endorsed the administration of oral misoprostol for PPH prevention by unskilled providers ‘trained in its use in settings where AMTSL is not practiced’ in its guidelines on prevention of PPH.^8^ The Royal College of Obstetricians and Gynaecologists in its recent PPH guidelines also recommends use of misoprostol when oxytocin is not available (for example, in a homebirth).^9^ Given that only one published randomised controlled trial has documented safe administration of misoprostol for PPH prevention when administered by trained TBAs in home delivery settings,^14^ there has been a call for additional evidence to support expanding misoprostol use for prevention of PPH in community settings and by lower level providers.^17^ The current trial sought to provide confirmatory evidence that 600 μg oral misoprostol is effective in preventing PPH and to demonstrate that TBAs can play the role of providers ‘trained in its use’.

## Methods

This double-blind randomised, placebo-controlled community-based trial evaluated the efficacy and safety of 600 μg oral misoprostol for the prevention of PPH in homebirth settings. The trial sought to test whether misoprostol reduces the incidence of PPH (≥500 ml) when administered by trained TBAs during the third stage of labour. The study was conducted in remote, mountainous villages of Chitral, Khyber Pakhtunkhwa Province, Pakistan where approximately half of all deliveries are conducted by an unskilled birth attendant at home. In this setting, the villages are situated at high elevations ranging from 1500 to 3500 m.

The TBAs, Lady Health Visitors (LHVs) and Community Health Nurses (CHNs), who were part of the Aga Khan Health Services, Pakistan (AKHS,P) network, were responsible for trial implementation. The study catchment area was limited to 46 villages surrounding 17 primary healthcare centres located within a driving distance of 2 hours from secondary-level facilities. Primary healthcare centres are staffed by LHVs and CHNs and provide basic maternal and child health services and obstetric care according to World Health Organization guidelines.^18^ The LHVs working for the AKHS,P centres have completed their matric grade ten in addition to 2 years of training midwifery and community health; CHNs have completed 3 years or more of training. Both LHVs and CHNs are responsible for training and supervision of TBAs and community health workers in the area. AKHS,P staff provide training to TBAs, which includes 15 days of initial instruction on safe delivery practices and referral procedures for women with complications. TBAs also participate in 3-day refresher trainings annually. A key component of their training is to keep centre-based staff informed of any pregnant women in the area, or when home delivery is imminent or has occurred.

Pregnant women in general good health, residing in one of the 46 study villages, and planning to deliver at home with a study TBA, were eligible for inclusion. Eligibility was confirmed by the LHV/CHN during antenatal care visit(s) and informed consent was obtained in the local language with signature or thumb impression. Iron folate tablets were given to women to take at the earliest antenatal visit possible as standard of care. Women were not eligible if presenting with pregnancy complications—such as hypertension, non-cephalic presentation, polyhydramnios, previous caesarean section, suspected multiple pregnancy, suspected still birth, antepartum haemorrhage, and Hb <8 g/dl. These women were referred for facility delivery. Eligible consenting women had finger-prick blood samples taken for Hb assessment during the last trimester of pregnancy, using a Hemocue® handheld device, which has a proven accuracy of ±1.5% compared with the international reference method for testing Hb (Hemocue, Ängelholm, Sweden). The Hemocue is a simple means of collecting Hb measures at the community-level where traditional laboratory techniques are not feasible. Women not attending antenatal care were identified by study TBAs and, if eligible, their consent was obtained and they were enrolled at delivery.

Participating women had their home deliveries managed by one of the 84 trained study TBAs. Immediately after delivery of the baby and before placental delivery, women were given by the study TBAs either three tablets of 200 μg misoprostol (GyMiso®; HRA Pharma, Paris, France) or matching placebo (resembling misoprostol) to take orally. Both women and TBAs were blinded to study assignment. The use of a placebo was considered ethical because the standard care for home deliveries with TBAs in the study area is to give no prophylactic uterotonic at delivery. Study medication was packed in numbered colour-coded boxes to identify the randomisation sequence. Study TBAs were provided with specially designed colour-coded drug boxes to ensure that the sequence was maintained. A computer-generated random code in blocks of six was maintained by Gynuity Health Projects in New York and not revealed until data collection and cleaning were completed.

Providers were asked to document how the third stage was managed for each participant. The TBAs are trained in management of the third stage of labour, including performing uterine massage, cord traction, delayed cutting of the cord, and immediate suckling at the breast. To collect postpartum blood loss, women were positioned on a perineal sheet and bedpan for a minimum of 1 hour or until active bleeding stopped—whichever occurred last. Study TBAs were provided with a 1-hour timer to track that blood was collected for 1 hour and were asked to estimate the time in minutes between delivery of the baby and of the placenta. Blood collected in the bedpan was transferred to a measuring jar, which was then closed, and the used perineal sheet and cotton roll were placed in a sealed plastic bag. The closed measuring jar and sealed plastic bag were then placed inside a plastic cooler which was tightly closed and stored in a secure place in the woman’s home until the LHV/CHN arrived for weighing, 1–2 days after delivery. If any complications were experienced at the time of delivery, the study TBA followed standard procedures; including performing uterine massage and arranging referral to a higher level of care. Generally, referrals involved having a skilled provider go to the woman’s home as opposed to transferring her to the health facility.

When a delivery occurred, the community health worker notified the facility-based staff, who then visited the woman at home within 24–48 hours to weigh the blood collected and interview the woman and TBA. At this visit, data were collected on any adverse effects or problems occurring after delivery. The LHV/CHN returned 3–5 days post-delivery to measure the woman's Hb.

Regular monitoring and training of study staff continued throughout the duration of the trial. The LHVs/CHNs conducted monthly follow-up visits with TBAs as part of routine practice. Study procedures and practices for managing deliveries were reviewed during these visits. The LHVs/CHNs were also visited on a monthly basis by their supervisors, at which time the study procedures were reviewed to ensure protocol adherence. Refresher trainings were held annually for all study staff.

**Figure 1:**
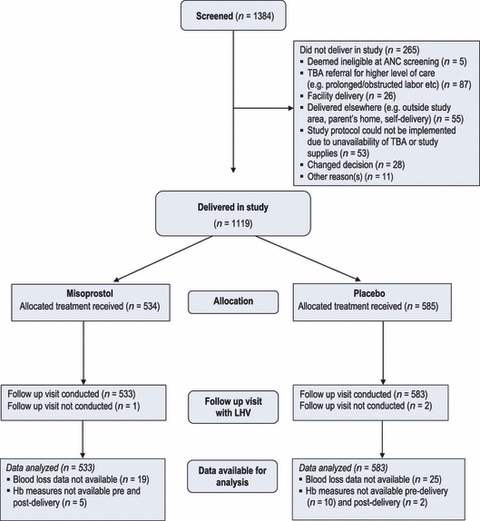
CONSORT flow diagram: trial profile.

The primary outcomes were PPH (defined as measured blood loss ≥500 ml) and drop in Hb > 2 g/dl. Secondary outcomes included intermediate and severe PPH (blood loss ≥750 and ≥1000 ml), mean blood loss and postpartum Hb <9 and <11 g/dl. To detect a 35% difference in the proportions of PPH (blood loss ≥500 ml) between the two study arms, a sample size of 543 was needed for a power of 80%, one-sided test and an alpha of 0.05 assuming that 15% of the placebo group had PPH. Assuming that the proportion of women experiencing a drop in Hb > 2 g/dl receiving placebo would be 20%, 470 women were required for the same alpha and power to detect a difference. The sample size was increased to 700 per arm to allow for up to 25% of screened women not delivering according to protocol. Characteristics of the two study arms were compared using chi-square or Fisher exact test for categorical variables and Mann–Whitney *U* test for continuous variables. Analysis of outcomes by year 1 and 2 was also conducted to explore whether improved delivery practices and study implementation influenced study outcomes. These subgroup analyses were not defined *a priori*. Data were double entered in Epi Windows (Centers for Disease Control, Atlanta, GA, USA) and later transferred into spss (SPSS, Chicago, IL, USA) and sas (SAS Institute Inc, Cary, NC, USA) for analysis. The protocol was approved by the Ethical Review Committee at the Aga Khan University (Karachi, Pakistan) and is reported in accordance with the revised CONSORT statement (Figure 1).^19^

## Results

During the initial 7 months of the study (25 October 2005 to 31 May 2006), 370 women were enrolled. A monitoring visit in May 2006 confirmed difficulties in using the blood collection and measurement tools, inaccuracies in recorded blood loss measures, and challenges with study monitoring during the winter months. This period of recruitment was therefore considered a pilot phase and all data collected during the initial 7 months have been excluded from the analysis. No safety issues were reported during the pilot phase. During the subsequent 2 years of the study, 1119 women were randomised to receive either 600 μg misoprostol orally (*n* = 534) or matching placebo (*n* = 585) during the third stage of labour from June 2006 to June 2008. In three deliveries, follow-up visits could not be carried out; data analysis was therefore conducted for 1116 women (misoprostol arm *n* = 533; placebo arm *n* = 583). With the exception of median pre-delivery Hb levels, baseline and delivery characteristics of women were similar (Table 1). All women received study medication per protocol and all outcomes were analysed by treatment assignment (per-protocol). Invalid blood loss measures, which mainly occurred when monitoring visits were not possible because of poor weather conditions, were excluded from our analysis (Figure 1).

**Table 1:** Baseline and delivery characteristics by study group*

	Misoprostol *n* = 533	Placebo *n* = 583
**Age (years)** mean (SD)	28 (5)	27 (4)
**Parity**		
Para 1	103 (19.3)	117 (20.1)
Para 2	142 (26.7)	134 (23.0)
Para 3–5	226 (42.4)	270 (46.3)
Para 6 or more	62 (11.6)	62 (10.6)
Pre-delivery Hb**		
Mean (SD)	12.7 (1.6)	12.9 (1.5)
Median (IQR)***	12.8 (11.7, 13.7)	13.0 (12.0, 14.0)
Range	8.2–18.0	8.2–16.8
Pre-delivery Hb < 9 g/dl**	5 (0.9)	6 (1.0)
Pre-delivery Hb < 11 g/dl**	73 (13.8)	65 (11.3)
**Number of ANC visits** median (IQR)	6 (4, 8)	6 (4, 8)
**Iron folate tablets taken during pregnancy**	455 (85.4)	485 (83.3)
**Number of months during pregnancy for which iron folate tablets were taken,** median (IQR)	3 (2, 4)	3 (2, 4)
**Highest level of school attended**	(*n* = 531)	(*n* = 580)
No formal education	386 (72.7)	397 (68.4)
Primary	53 (10.0)	79 (13.6)
Secondary and above	92 (17.3)	99 (17.0)
**Woman’s occupation** (multiple responses possible)		
Housewife	527 (98.9)	573 (98.3)
Farmer	92 (17.3)	78 (13.4)
Professional	9 (1.7)	13 (2.2)
**Delivery characteristics**		
Baby born alive	528 (99.1)	575 (98.6)
Nipple stimulation performed by TBA at delivery	118 (22.1)	111 (19.0)
Uterine massage performed by TBA at delivery	389 (73.0)	423 (72.6)
Cord traction performed by TBA at delivery	151 (28.3)	168 (28.8)
Time (minutes) between delivery of baby and placenta as estimated by TBAs, median (IQR)	20 (10, 30)	15 (10, 30)
Placental delivery within 30 minutes of delivery of baby	368 (69.0)	418 (71.7)

IQR, interquartile range.

* Numbers are *n* (%) unless otherwise specified.

** Predelivery haemoglobin measures available for 528 women in misoprostol group and 573 women in placebo group.

*** Mann–Whitney *U* test confirms statistical significance with two-tailed *P*-value of 0.043.

Data on blood loss after delivery were available for 514 women who received misoprostol and 558 who received placebo. The primary outcome measure of blood loss ≥500 ml shows that women who received misoprostol had a lower rate of PPH (16.5%) compared with 21.9% for those given placebo (RR 0.76, 95% CI 0.59–0.97) (Table 2). Median total blood loss after delivery was comparable among the two study groups (*P* = 0.103). Ten women in the misoprostol group had blood loss ≥1000 ml compared with 19 women in the placebo group; however, this difference between arms did not reach statistical significance. For every 19 women given misoprostol prophylactically, one incidence of PPH (blood loss ≥500 ml) was averted.

**Table 2:** Blood loss and haemoglobin (Hb) outcomes by study group*

	Misoprostol	Placebo	Relative risk (95% CI)
**Primary outcomes**			
Blood loss ≥500 ml**	85/514 (16.5)	122/558 (21.9)	0.76 (0.59–0.97)
Drop in Hb > 2 g/dl***	88/528 (16.7)	120/572 (21.0)	0.79 (0.62–1.02)
**Secondary outcomes**			
Blood loss	(*n* = 514)	(*n* = 558)	
Blood loss ≥750 ml	29/514 (5.6)	40/558 (7.2)	0.79 (0.50–1.25)
Blood loss ≥1000 ml	10/514 (1.9)	19/558 (3.4)	0.57 (0.27–1.22)
Total blood loss (ml)			
Median (IQR)	280 (200, 400)	300 (200, 460)	–
Mean (SD)	337 (226)	366 (262)	
Range	0–1820	20–1890	
Haemoglobin	(*n* = 533)	(*n* = 581)	
Post-delivery Hb			
Median (IQR)	11.6 (10.5, 12.8)	11.6 (10.5, 12.8)	–
Mean (SD)	11.6 (1.6)	11.5 (1.6)	
Range	(5.4–15.8)	(5.0–16.0)	
Post-delivery Hb < 9 g/dl	27/533 (5.1)	37/581 (6.4)	0.80 (0.49–1.29)
Post-delivery Hb < 11 g/dl	182/533 (34.1)	189/581 (32.5)	1.05 (0.89–1.24)
Change in Hb***			
Median (IQR)	1.0 (0.5, 1.7)	1.2 (0.5, 1.9)	–
Mean (SD)	1.1 (1.2)	1.3 (1.4)	
Drop in Hb > 3 g/dl***	27/528 (5.1)	55/572 (9.6)	0.53 (0.34–0.83)

* Numbers are *n* (%) unless otherwise specified.

** Blood measures available for 514 women in the misoprostol group and 558 women in the placebo group.

*** Pre- and post-delivery Hb measures available for 528 women in the misoprostol group and 572 women in the placebo group for calculation of change/drop in Hb levels from pre- to post-delivery.

Analysis of Hb exhibits a similar pattern of blood loss (Table 2). A change in Hb levels >2 g/dl, pre- to post-delivery, was experienced by 16.7% of women given misoprostol, compared with 21.0% in the placebo group (RR 0.79, 95% CI 0.62–1.02). An analysis of drop in Hb > 3 g/dl shows that Hb decreases were statistically different between study arms (misoprostol 5.1% versus placebo 9.6%, RR 0.53, 95% CI 0.34–0.83). A smaller, yet clinically insignificant change in Hb pre- to post-delivery was found in the misoprostol group, with a median drop of 1.0 g/dl compared with 1.2 g/dl with placebo (*P* = 0.016). Haemoglobin outcomes were also analysed for the subset of women who did not take iron folate medication during pregnancy (misoprostol group = 78; placebo group = 98). Analyses of the change in Hb concentrations pre- to post-delivery (>2 and >3 g/dl) confirmed that findings were similar to those for the entire cohort (data not shown).

Shivering and chills were the most commonly reported adverse effects (Table 3); occurring for one of every ten women receiving misoprostol and one of every 20 women given placebo. Four cases of fever were detected in the misoprostol group versus seven in the placebo group; and all other adverse effects were minimal. There were no maternal deaths or any other serious adverse events reported during the trial. Retained placenta and PPH were the most common reasons identified by the TBA for referral to higher level of care (Table 3). There were no differences between study groups in the proportion of women receiving higher-level care (1.7% in misoprostol group versus 1.7% in placebo group).

**Table 3:** Adverse effects and referrals by study group*

	Misoprostol *n* = 533	Placebo *n* = 583	*P*-value**
**Adverse effects reported**			
Nausea	8 (1.5)	8 (1.4)	0.527
Vomiting	3 (0.6)	3 (0.5)	0.614
Diarrhoea	1 (0.2)	0 (0.0)	0.478
Shivering	50 (9.4)	23 (3.9)	<0.0001
Chills/cold	53 (9.9)	29 (5.0)	<0.0001
Fever	4 (0.8)	7 (1.2)	0.326
Headache	6 (1.1)	7 (1.2)	0.566
Weakness/fatigue	9 (1.7)	8 (1.4)	0.425
Dizziness/fainting	9 (1.7)	6 (1.0)	0.244
**Referrals**			
Woman referred for higher level of care			
Multiple reasons possible	9 (1.7)	10 (1.7)	0.579
Due to PPH	2 (0.4)	3 (0.5)	0.542
Due to retained placenta	7 (1.3)	7 (1.3)	0.538
**Maternal death**	0 (0.0)	0 (0.0)	–

* Numbers are *n* (%) unless otherwise specified.

** One-tailed *P*-values are specified.

**Figure 2:**
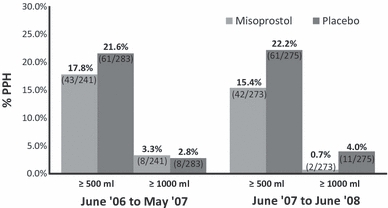
Rates of PPH (≥500 and ≥1000 ml) for two sequential subgroups of women randomised to receive misoprostol or placebo.

**Figure 3:**
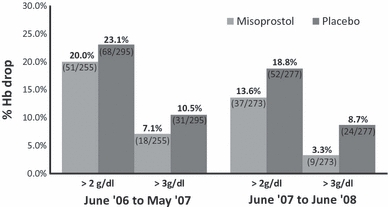
Rates of Hb drop pre- to post-delivery (>2 and >3 g/dl) for two sequential subgroups of women randomised to receive misoprostol or placebo.

Figures 2 and 3 present the rates of PPH (≥500 and ≥1000 ml) and of Hb concentration changes (>2 and >3 g/dl) over the course of the study. In the first year of the study, the effect of misoprostol on PPH rates and changes in Hb was negligible, whereas in the following year, misoprostol was associated with a significant reduction in the rates of PPH and in the proportion of women who experienced a Hb drop >3 g/dl, compared with placebo (PPH: RR 0.69, 95% CI 0.49–0.99; severe PPH: RR 0.18, 95% CI 0.04–0.82; Hb drop >3 g/dl: RR 0.38, 95% CI 0.18–0.80). Analysis of the temporal trends within each study arm confirmed no statistically significant reductions in rates of PPH or in Hb changes in the placebo group. In the misoprostol group, 3.3% of women had severe PPH in the first year, compared with 0.7% in the second year (RR 0.26, 95% CI 0.06–1.21). Similar trends were observed for drops in Hb >2 and >3 g/dl in the misoprostol group (respective RR 0.68, 95% CI 0.46–1.00; RR 0.47, 95% CI 0.21–1.02).

## Discussion

This randomised controlled trial shows that 600 μg oral misoprostol confers benefit in the reduction of PPH among women delivering at home with a trained TBA. Oral misoprostol reduced the rate of PPH (≥500 ml) by 24% compared with placebo. These findings corroborate published evidence on the efficacy of misoprostol for PPH prevention when used for home deliveries and in primary-care centres.^14^–^16^ A placebo-controlled community-based trial conducted in India showed a 50% reduction in PPH ≥ 500 ml with misoprostol. The trial in India, as well as a trial conducted in Guinea Bissau, also documented that misoprostol reduced the incidence of severe haemorrhage (≥1000 ml) by 80% and 34%, respectively.^15^,^16^ In comparison, the findings presented in this paper show a statistically significant reduction in severe bleeding by 82%, but only among women who delivered in the second year of the study. Although misoprostol’s effect on blood loss has largely been consistent across placebo-controlled trials objectively measuring blood loss, differences in the magnitude of effect have been noted.^11^ Variation in delivery practices during the third stage of labour are postulated as possibly contributing to these differences, as providers in some trials practiced AMTSL whereas others practiced ‘passive’ management.^11^ Irrespective of these differences, this trial strengthens the evidence base for justifying use of misoprostol during routine third stage of labour management when conventional injectable uterotonics are not feasible.

An unanticipated finding of this study relates to the unusually high level of PPH documented in this population, which averaged 19% among the study arms. In contrast with other community-based trials that compared 600 μg oral misoprostol with placebo or ergometrine, PPH rates (≥500 ml) averaged 9 and 11%, respectively.^14^,^16^ High blood loss outcomes in this setting may possibly be attributed to the high elevation and higher haemoglobin concentrations. Interestingly, a hospital-based study measuring blood loss conducted in Llasa, Tibet (elevation 3650 m) also documented a rate of PPH (≥500 ml) that averaged 15% between study arms comparing a similar misoprostol regimen given prophylactically with a Tibetan traditional medication administered during the third stage of labour.^20^

The present study also showed that significantly fewer women in the misoprostol group experienced a drop in Hb > 3 g/dl compared with women given placebo. This finding concurs with previous research that exhibited a protective effect of misoprostol on Hb levels when compared with ergometrine or placebo.^14^,^21^,^22^ The clinical relevance of misoprostol’s protective effect is critical, given that PPH and anaemia contribute independently and interactively to a large proportion of adverse maternal outcomes. To this end, some guidelines note that pre- and post-delivery Hb levels should be taken into consideration when diagnosing cases of PPH and providing follow-up care.^9^,^23^ In high elevation settings, where Hb concentrations are notably higher, altitude-specific haemoglobin cut-offs for defining iron deficiency deserve attention.^24^ This study was designed to compare the proportions of women with Hb levels <9 and <11 g/dl between study groups. However, the number of women with Hb levels below these pre-specified cut-offs was quite low, and may not adequately reflect iron deficiency in this population.

Shivering and fever are common adverse effects of misoprostol; but adverse effects in this trial were relatively low. Transient shivering and chills were experienced by 10% of women receiving misoprostol versus 5% in the placebo group. All other adverse effects, including fever, were minimal, and their occurrence did not differ between study arms. In previous PPH prevention studies, rates of shivering have been reported in as few as 19% of women following 600 μg oral misoprostol, and as many as 62%.^25^ Isolated reports of transient fever above 40°C have also been documented in two trials testing a similar regimen for PPH prevention.^10^,^26^ Previous reports of high fever following a 600 μg regimen of oral misoprostol include five of 9198 and four of 1026 women. None resulted in any complication.^10^,^26^ The low rates of adverse effects documented in this study may be the result of recall bias by the woman or TBA, as data collection on adverse effects did not occur at the time of delivery but at the LHV’s follow-up visit to the woman’s home 1–2 days postpartum. Body temperature was not systematically measured after administration of study medication.

The trial does have some limitations. Primarily, the outcomes of this study may not be generalisable to all rural settings. This trial was conducted in collaboration with AKHS,P, capitalising on the extensive network between AKHS,P providers and trained TBAs, with functioning systems already in place for handling referrals. A second limitation stems from the difficulties in validating blood loss measurements during the initial phase of the study when monitoring visits were not possible because of poor weather conditions. The first external monitoring visit in May 2006 revealed that the study team were incorrectly using the blood assessment tools to collect, weigh and read the scale and that additional training and more frequent monitoring was needed. After a careful review of all data collected to that point, the study team determined that measures of blood collected from October 2005 through May 2006 could not be analysed because they were invalid. Nonetheless, the sample size was met by extending the enrolment period. The exclusion of blood loss measures during the first phase of the trial resulted in an underpowered arm for analysing the primary outcome of PPH (≥500 ml) and disproportionate samples for comparing the two study groups. The difficulties that were encountered highlight the importance of conducting pilot studies to ensure correct implementation of all study procedures.

A major strength of the trial is that rigorous data collection was ultimately possible in a remote setting, with TBAs playing a major role in study implementation. Trained TBAs, who were mostly illiterate, proved able to safely and correctly follow instructions regarding the administration of misoprostol after delivery of the baby, collect blood loss, and manage referrals in a timely manner. With adequate training and supervision provided by AKHS,P, there were no safety issues in this study, and TBAs were able to recognise and arrange prompt referrals for complications of delivery. The temporal trends in Figures 2 and 3 underscore the importance of continual support, training, and skill-building. In the second year of the study, misoprostol was associated with significant reduction in postpartum bleeding and in the proportion of women who experienced clinically important changes in haemoglobin concentrations; whereas the first year did not produce any measurable differences between the two study arms. These trends suggest that other factors besides uterotonic potency, such as improved implementation, training and delivery skills, may have contributed to misoprostol’s measurable effect on PPH prevention in the second year of the study. In fact, an analysis of other outcome variables shows significant improvements in both antenatal and delivery care in the last year of the study, in comparison with the first year (data not shown).

Based on a 2006 technical consultation on the prevention of PPH, the World Health Organization recommends that in the absence of AMTSL uterotonic drugs be offered by health workers trained in their use.^8^ In this document, the WHO notes “For misoprostol, this recommendation places a high value on the benefits of avoiding PPH and the ease of administration of an oral drug in settings in which other care is not available.”^8^ This study adds to the evidence that misoprostol can be administered safely by trained TBAs and is effective in reducing PPH when other active management components are practiced.^14^ Homebirth remains the strong preference, and often the only option, for many women in the developing world. A large proportion of these births take place without skilled birth attendants: the total number is estimated at 60 million annually. To train the 400 000 midwives needed to cover these deliveries, as well as finding the required salaries, housing and allowances for postings in rural areas, work opportunities for their spouses and educational facilities for their children, will take time.^27^ Misoprostol administration by health workers trained in its use for the prevention of PPH can have immediate benefits.

There are some unanswered questions related to misoprostol use for PPH prevention. For instance, current evidence supports a 600 μg oral dose, yet it is conceivable that 400 μg could also be effective. A recent meta-analysis found no benefit of 600 over 400 μg misoprostol for blood loss ≥1000 ml (RR 1.02, 95% CI 0.71–1.48).^28^ A lower dose may decrease the adverse effects experienced associated with misoprostol. As more evidence becomes available on lower doses of misoprostol for PPH prevention, dosage recommendations should be re-evaluated. Additional research may also be needed to document the effectiveness of large service delivery programmes offering misoprostol for PPH prevention and to better understand whether postpartum misoprostol administration will save lives. Furthermore, to achieve the United Nation’s Millennium Development Goal #5 to improve maternal health, urgent action and investment are needed to accelerate progress for coverage of clinical-care interventions, which depend on adequate access, human resources and essential supplies.^29^

These findings provide additional evidence for the role of misoprostol in reducing PPH when administered by a trained TBA. Given that, for now, misoprostol may be the only feasible PPH prevention option, it should be endorsed as a safe and effective alternative intervention for use at home deliveries.

## References

[1] Khan KS, Wojdyla D, Say L, Gülmezoglu AM, Van Look PFA (2006). WHO analysis of causes of maternal death: a systematic review. Lancet.

[2] Ramanathan G, Arulkumaran S (2006). Postpartum haemorrhage. J Obstet Gynaecol Can.

[3] AbouZahr C (2003). Global burden of maternal death and disability. Br Med Bull.

[4] United Nations (2009). The millennium development goals report 2009 [Online]. http://www.un.org/millenniumgoals/pdf/MDG%20Report%202009%20ENG.pdf.

[5] National Institute of Population Studies (NIPS) [Pakistan], Macro International Inc (2008). Pakistan Demographic and Health Survey 2006–07.

[6] Prendiville WJ, Harding JE, Elbourne DR, Stirrat GM (1988). The Bristol third stage trial: active versus physiological management of third stage of labour. Br Med J.

[7] International Confederation of Midwives; International Federation of Gynaecologists and Obstetricians (2004). Joint statement: management of the third stage of labour to prevent postpartum haemorrhage. J Midwifery Womens Health.

[8] WHO DoMPS (2007). WHO Recommendations for the Prevention of Postpartum Haemorrhage.

[9] Royal College of Obstetricians and Gynaecologists (2009). Prevention and Management of Postpartum Haemorrhage.

[10] Gülmezoglu AM, Villar J, Ngoc NT, Piaggio G, Carroli G, Adetoro L (2001). WHO multicentre randomised study of misoprostol in the management of the third stage of labour. Lancet.

[11] Gülmezoglu AM, Forna F, Villar J, Hofmeyr G (2007). Prostaglandins for preventing postpartum haemorrhage. Cochrane Database Syst Rev.

[12] Alfirevic Z, Blum J, Walraven G, Weeks A, Winikoff B (2007). Prevention of postpartum haemorrhage with misoprostol. Int J Gynecol Obstet.

[13] Tsu VD, Shane B (2004). New and underutilized technologies to reduce maternal mortality: call to action from a Bellagio workshop. Int J Gynecol Obstet.

[14] Walraven G, Blum J, Dampha Y, Sowe M, Morison L, Winikoff B (2005). Misoprostol in the management of the third stage of labour in the home delivery setting in rural Gambia: a randomised controlled trial. Br J Obstet Gynaecol.

[15] Høj L, Cardoso P, Nielsen BB, Hvidman L, Nielsen J, Aaby P (2005). Effect of sublingual misoprostol on severe postpartum haemorrhage in a primary health centre in Guinea-Bissau: randomised double blind clinical trial. Br Med J.

[16] Derman RJ, Kodkany BS, Goudar SS, Geller SE, Naik VA, Bellad MB (2006). Oral misoprostol in preventing postpartum haemorrhage in resource-poor communities: a randomised controlled trial. Lancet.

[17] WHO (2009). Unedited draft report of the 17th Expert committee on the selection and use of essential medicines, 23 to 27 March 2009. Unedited WHO technical report series (version 18 May 2009) [Online]. http://www.who.int/selection_medicines/committees/expert/17/WEBuneditedTRS_2009.pdf.

[18] WHO, Division of Family Health, Maternal Health and Safe Motherhood Programme (1994). Care of Mother and Baby at the Health Centre: A Practical Guide.

[19] Altman DG, Schulz KF, Moher D, Egger M, Davidoff F, Elbourne D (2001). The revised CONSORT statement for reporting randomized trials: explanation and elaboration. Ann Intern Med.

[20] Miller S, Tudor C, Thorsten V, Nyima, Kalyang, Sonam (2009). Randomized double masked trial of Zhi Byed 11, a Tibetan traditional medicine, versus misoprostol to prevent postpartum hemorrhage in Lhasa, Tibet. J Midwifery Womens Health.

[21] Enakpene CA, Morhason-Bello IO, Enakpene EO, Arowojolu AO, Omigbodun AO (2007). Oral misoprostol for the prevention of primary post-partum hemorrhage during third stage of labor. J Obstet Gynaecol Res.

[22] Surbek DV, Fehr PM, Hösli I, Holzgreve W (1999). Oral misoprostol for third stage of labor: a randomized placebo-controlled trial. Obstet Gynecol.

[23] ACOG Practice Bulletin (2006). Clinical management guidelines for obstetrician-gynaecologists number 76, October 2006: postpartum haemorrhage. Obstet Gynecol.

[24] Cohen JH, Haas JD (1999). Hemoglobin correction factors for estimating the prevalence of iron deficiency anemia in pregnant women residing at high altitudes in Bolivia. Rev Panam Salud Publica.

[25] Patted SS, Goudar SS, Naik VA, Bellad MB, Edlavitch SA, Kodkany BS (2009). Side effects of oral misoprostol for the prevention of postpartum haemorrhage: results of a community-based randomised controlled trial in rural India. J Matern Fetal Neonatal Med.

[26] Ng PS, Chan ASM, Sin WK, Tang LCH, Cheung KB, Yuen PM (2001). A multicentre randomised controlled trial of oral misoprostol and i.m. syntometrine in the management of the third stage of labor. Hum Reprod.

[27] Walraven G, Weeks A (1999). The role of (traditional) birth attendants with midwifery skills in the reduction of maternal mortality. Trop Med Int Health.

[28] Hofmeyr JG, Gülmezoglu AM (2008). Misoprostol for the prevention and treatment of postpartum haemorrhage. Best Pract Res Clin Obstet Gynaecol.

[29] Bryce J, Daelmans B, Dwivedi A, Fauveau V, Countdown Coverage Writing Group, Countdown to 2015 Core Group (2008). Countdown to 2015 for maternal, newborn, and child survival: the 2008 report on tracking coverage of interventions. Lancet.

